# Genomic characteristics and phylogenetic analyses of colonization and infection with carbapenem-resistant *Klebsiella pneumoniae* in multicenter intensive care units: a cohort study

**DOI:** 10.1128/spectrum.01584-24

**Published:** 2025-02-24

**Authors:** Yi-Le Wu, Wen-Wen Chu, Xiao-Qian Hu, Yi-Yu Lyu, Jie-Hao Tai, Ruo-Jie Li, Kai Huang, Xue Zhao, Wen-Hui Zhang, Xue-Ping Wang, Xiang Yan, Zhou Liu, Xi-Yao Yang, Qiang Zhou, Min Yang

**Affiliations:** 1 Department of Hospital Infection Prevention and Control, the Second Affiliated Hospital of Anhui Medical University 533251 , Hefei, Anhui, China; 2 Department of Clinical Laboratory, the Second Affiliated Hospital of Anhui Medical University, Hefei, Anhui, China; 3 The Fourth Affiliated Hospital of Anhui Medical University, Hefei, Anhui, China; 4 Nursing Department, the Second Affiliated Hospital of Anhui Medical University, Hefei, Anhui, China; 5 The Second Department of Critical Care Medicine, the Second Affiliated Hospital of Anhui Medical University, Hefei, Anhui, China; Laboratory Corporation of America Holdings, Burlington, North Carolina, USA

**Keywords:** carbapenem resistance, *Klebsiella pneumoniae*, whole-genome sequencing, intensive care unit, prospective

## Abstract

Carbapenem-resistant *Klebsiella pneumoniae* (*CRKp*) poses a major threat to global public health. This study aimed to investigate the genomic characteristics and phylogenetic relatedness of colonization and infection with *CRKp* among intensive care unit (ICU) patients. A total of 61 *CRKp* isolates, including 29 non-ICU-acquired and 32 ICU-acquired strains, were collected by active screening and infection culture from 16 ICUs through a multicenter cohort study. Following whole-genome sequencing (WGS), seven sequence types (STs) were identified, with ST11 (60.7%) being the most dominant, followed by ST15 (27.9%). Most strains (93.4%) carried the *bla*KPC-2 gene, while 9.8% and 3.3% of strains carried either the *bla*NDM-1 gene or both genes. A high abundance of virulence genes including *iutA* (55.7%), *rmpA* (18.0%), and *rmpA2* (52.5%) were identified, with *iutA +rmpA2* (37.7%) being the most common combination. The IncHI1B plasmid was identified in all of the 34 strains carrying the detected virulence genes. Furthermore, results from cgMLST analysis revealed 10 clusters with highly homogeneous *CRKp* strains, from which the potential interregional and intrahospital spread of *CRKp* were hypothesized. Here, this study reveals the widespread distribution of *CRKp* as well as locally specific strains in different regions of Anhui Province. The observed high abundance of virulence genes requires additional attention and continued monitoring. Taken together, these findings highlight the need for coordinated efforts between healthcare facilities and networks to aid *CRKp* control strategies and prevent spread. Moreover, we emphasize the importance of WGS in revealing additional insights, thus improving epidemiological studies and transmission control of *CRKp*.

**IMPORTANCE:**

Carbapenem-resistant *K. pneumoniae* (*CRKp*) has spread rapidly to different parts of the world and poses a serious threat to global health. High genetic diversity in *CRKp* can introduce complexities in disease treatment and management. Intensive care unit (ICU) patients are more susceptible to acquire *CRKp* infections. However, most *CRKp* studies have focused on strains isolated from infections, rather than cases of asymptomatic *CRKp* colonization. This study analyzed clinical *CRKp* isolates from ICU patients as well as isolate cases of active colonization screening. Findings reveal the genetic diversity of *CRKp* in different regions of Anhui Province, emphasizing the necessity for a more comprehensive investigation of the genomic characteristics and phylogenetic relatedness of *CRKp* in different regions. Data regarding antimicrobial resistance genes, virulence genes, and genetic relatedness will improve the understanding of the potential risk of *CRKp* to public health and aid guidance for prevention and control of *CRKp*.

## INTRODUCTION

*Klebsiella pneumoniae* is a common pathogen of community- and hospital-acquired infections that can adapt considerably in different habitats (1). Although *K. pneumoniae* is commonly found as a member of the human gut microflora, it can acquire plasmids harboring carbapenemase genes, leading to the emergence of superbug “carbapenem-resistant *K. pneumoniae* (*CRKp*),” which causes 33%–50% mortality rates of infections worldwide (2, 3). *CRKp* first emerged in the United States in the early 2000s and has spread rapidly to different parts of the world, and it has been listed as one of the highest-priority pathogens by the World Health Organization (WHO) (4, 5). According to data from the China Antimicrobial Surveillance Network (CHINET), *CRKp* prevalence increased rapidly from 6.4% in 2014 to 11.3% in 2021 (6). A concerning *CRKp* epidemic was reported in Anhui Province in 2021, during which time the detection rate was approximately 15%, ranking fourth in China (6). Therefore, there is an urgent need to implement effective control strategies to prevent a global *CRKp* pandemic.

There is evidence in the literature indicating high genetic diversity in *CRKp* (7, 8). Despite the multiple mechanisms that account for carbapenem resistance in *CRKp*, carbapenemase production is the most common and clinically relevant (4). *K. pneumoniae* carbapenemase (KPC) is the most prevalent, and several other genes that encode carbapenemases, including the New Delhi metallo-β-lactamase (NDM), Verona integron-encoded metallo-β-lactamase (VIM), Oxacillinase-48-type carbapenemases (OXA-48), and Imipenemase metallo-β-lactamase (IMP), are common in China (9, 10). Sequence type (ST) 258 and ST11 have been identified as the most common STs in *CRKp* strains in different parts of the world. Furthermore, ST258 has spread globally and has become particularly prevalent in North America, Latin America, and European countries, while ST11 is the dominant clone in China (11). In addition, the prevalence of hypervirulent *CRKp* strains harboring virulence genes has increased rapidly in China in recent years. Importantly, some hypervirulent *CRKp* strains pose major threats to public health because they are highly transmissible, multidrug resistant, as well as hypervirulent (12). These factors can complicate the treatment and clinical management of *CRKp*.

Intensive care unit (ICUs) patients are more susceptible to acquire *CRKp* (13), which is associated with multiple factors, including antibiotic exposure, frequent invasive treatments, and a longer duration of hospitalization (14, 15). Research has shown that rectal colonization with *CRKp* occurs frequently in ICU patients (13), and colonization with *K. pneumoniae* is a major risk factor for infection (16). Because asymptomatic *CRKp* is difficult to diagnose, the majority of *CRKp* strains are typically isolated from symptomatic infected patients. As a result, individuals with asymptomatic infections may be considerable sources of infection and transmission (17). Comprehensive surveillance of colonization and infection with *CRKp* would be beneficial for informing epidemic trends, guiding treatment selection and public health policies, as well as assessing the impact of different interventions (9, 18). Previous studies have also described differences in clinical and microbiological features between community- and hospital-acquired *CRKp* (19). Therefore, there is a need for a comprehensive investigation of the genomic characteristics of ICU-acquired and non-ICU-acquired *CRKp*.

Herein, this multicenter cohort study applying whole-genome sequencing (WGS) aimed to identify antimicrobial resistance genes and virulence genes, as well as to characterize the phylogenetic relatedness of ICU-acquired and non-ICU-acquired *CRKp* colonization and infection collected from patients admitted to ICUs in Anhui Province, China. These data will improve the understanding of the epidemiology of *CRKp* in ICUs and provide a basis for targeted treatment, as well as prevention and control strategies.

## MATERIALS AND METHODS

### Study design and patient population

Samples and data were obtained from a prospective multicenter cohort study that was performed from 1st December 2020 to 31st January 2021 in comprehensive ICUs of six provincial, 15 municipal, and three district hospitals in Anhui Province, China (20). In the cohort study, adult patients who were newly admitted to the ICU wards were potentially eligible. However, patients with an ICU stay ≤48 h or incomplete clinical or microbiological data were excluded from the cohort.

### CRKp isolates and data collection

The *CRKp* strains were collected from both active colonization screening and clinical isolates from various sources of infections. *CRKp* colonization was screened via rectal swab samples from cohort patients admitted to the ICU within 48 h of admission and discharge, respectively. Chromogenic-based selective agar (CHROMagar, France) was used for the initial screening of *CRKp*, with the growth of blue bacterial colonies on the agar after 24 h of culture being suggestive of *CRKp*. Bacterial strains obtained from enrolled patients with clinical symptoms of infections were also collected to determine the presence of *CRKp*. All suspected strains were further analyzed for species identification by mass spectrometry (Bruker Autoflex MALDI-TOF MS, Germany). Susceptibility tests of carbapenem resistance for meropenem, imipenem, and ertapenem were performed using the VITEK 2 AST-XN04 and AST-N334 cards on VITEK-2 Compact instrument fully automated microbial drug sensitivity analyzer (bioMérieux, Marcy L'Étoile, France), and minimal inhibitory concentration values were determined. These results were interpreted according to the standards of the Clinical and Laboratory Standards Institute guidelines. Carbapenem resistance was defined as resistance to one or more carbapenems (21). In total, 61 non-duplicated *CRKp* strains were collected and recovered from 16 comprehensive ICUs in nine cities.

In this study, ICU-acquired *CRKp* was defined as a culture from a colonization or clinical specimen more than 48 h after ICU admission referring to the Diagnostic Criteria for Nosocomial Infection in China (22), and these criteria were modified based on criteria from the Center for Disease Control and Prevention (23). Whereas non-ICU-acquired *CRKp* is considered a culture of colonization or infection taken within 48 h after the ICU admission. Demographic and clinical data were collected from electronic medical records.

### DNA extraction and WGS analysis

Genomic DNA from *CRKp* strains was extracted using the cetyltrimethylammonium bromide (CTAB) method with slight modifications (24). The concentration, quality, and integrity of the extracted DNA were assessed using a Qubit fluorometer (Invitrogen, USA) as well as a NanoDrop spectrophotometer (Thermo Scientific, USA). Sequencing libraries of the strains were generated using the TruSeq DNA Sample Preparation Kit (Illumina, USA) and the Template Prep Kit (Pacific Biosciences, USA). Genome sequencing was carried out by the Personal Biotechnology Company (Shanghai, China) using the Illumina Novaseq platform (Illumina, USA). After adapter contamination removal and data filtering using the AdapterRemoval (25) and SOAPec (26) tools, the filtered reads were assembled to construct scaffolds and contigs using SPAdes (27) and A5-miseq (28). Finally, all of the assembled genomes were integrated to generate a complete sequence, and the genome sequence was acquired after rectification using the Pilon software (29). The average sequence length of these 61 strains was 5,682,889.84 bp, ranging from 5,363,359 to 5,970,300 bp.

### Analysis of sequence typing, antimicrobial genotypes, and virulence factors

Multilocus sequence typing (MLST) was performed to determine STs using the MLST 2.0 (https://cge.food.dtu.dk/services/MLST/). Capsule serotyping analyses were performed using the Kaptive Web tool (https://github.com/katholt/Kaptive), and resistance genes were identified using ResFinder (http://genepi.food.dtu.dk/resfinder). Considering recent reports (30, 31), a set of representative virulence genes, including the regulator of the mucoid phenotype (*rmpA*), the regulator of mucoid phenotype 2 (*rmpA2*), and aerobactin (*iutA*) were identified using the Virulence Factor Database (http://www.mgc.ac.cn/cgi-bin/VFs/v5/main.cgi), and IncHI1B plasmid was analyzed using PlasmidFinder (https://cge.food.dtu.dk/services/PlasmidFinder/).

### Single nucleotide polymorphism analysis and phylogenetic analysis

Genome sequences of all strains were submitted to Pathogenwatch platforms (https://pathogen.watch/), which was developed as an analytics tool for genomic and epidemiological data of *Klebsiella* species (32), to construct an SNP-based phylogenetic tree based on the neighbor-joining method. The phylogenetic tree was visualized using iTOL software (http://itol2.embl.de). SeqSphere + version 10.0 (Ridom, Münster, Germany; https://www.ridom.de/news/) and the available core genome MLST (cgMLST) scheme comprising 2,358 target genes for *K. pneumoniae* were applied to prepare the minimum spanning tree, and the preliminary cutoff of close relatedness was defined with ≤10 allele differences referencing a previous study (33).

### Statistical analysis

Comparisons involving categorical variables between the ICU-acquired and non-ICU-acquired *CRKp* groups were tested using the Chi-square tests (*χ*^2^) or Fisher’s exact tests, as appropriate. All the statistical analyses were performed using SPSS 26.0 software (SPSS, Inc., Chicago, IL, USA). A two-tailed *P* value < 0.05 was considered to indicate statistical significance.

## RESULTS

### Epidemiological and clinical data

Among the 61 *CRKp* strains, 29 (47.5%) were classified as non-ICU-acquired *CRKp* and 32 (52.5%) were classified as ICU-acquired *CRKp* (Table S1). Compared with ICU-acquired *CRKp* strains, non-ICU-acquired *CRKp* strains were significantly more likely to be isolated from ICU patients who were transferred from other hospitals (*P* = 0.001), had been admitted to a hospital (*P* = 0.013) or to an ICU within 1 year (*P* < 0.001), and had received any antibiotics (*P* = 0.048) or fluoroquinolones within the past 3 months (*P* = 0.046) (Table 1).

**TABLE 1:** Demographic and clinical information of 61 patients with *CRKp*

Variables	Total (*N* = 61)	Non-ICU-acquired *CRKp* (*n* = 29)	ICU-acquired *CRKp* (*n* = 32)	P
Study facility				0.600
Provincial/municipal hospitals	58 (95.1%)	27 (93.1%)	31 (96.9%)	
District hospitals	3 (4.9%)	2 (6.9%)	1 (3.1%)	
Age (year)				0.689
<45	6 (9.8%)	3 (10.3%)	3 (9.4%)	
45–59	16 (26.2%)	9 (31.0%)	7 (21.9%)	
≥60	39 (63.9%)	17 (58.6%)	22 (68.8%)	
Sex				0.306
Male	38 (62.3%)	20 (69.0%)	18 (56.3%)	
Female	23 (37.7%)	9 (31.0%)	14 (43.8%)	
Comorbid conditions				
Hypertension	36 (59.0%)	15 (51.7%)	21 (65.6%)	0.270
Diabetes mellitus	15 (24.6%)	6 (20.7%)	9 (28.1%)	0.501
Trauma	5 (8.2%)	3 (10.3%)	2 (6.3%)	0.662
Infectious diseases	21 (34.4%)	11 (37.9%)	10 (31.3%)	0.652
Cardiovascular/cerebrovascular diseases	45 (73.8%)	23 (79.3%)	22 (68.8%)	0.349
Neoplasms	2 (3.3%)	0 (0.0%)	2 (6.3%)	0.493
Respiratory diseases	14 (23.0%)	6 (20.7%)	8 (25.0%)	0.698
Blood disorders	2 (3.3%)	1 (3.4%)	1 (3.1%)	1.000
Neurological diseases	3 (4.9%)	2 (6.9%)	1 (3.1%)	0.600
Renal diseases	5 (8.2%)	2 (6.9%)	3 (9.4%)	1.000
Gastrointestinal diseases	6 (9.8%)	2 (6.9%)	4 (12.5%)	0.674
Transfer from other hospital				0.001
Yes	8 (13.1%)	8 (27.6%)	0 (0.0%)	
No	53 (86.9%)	21 (73.3%)	32 (100.0%)	
Healthcare exposures within 1 year				
Admitted to a hospital	43 (70.5%)	25 (86.2%)	18 (56.3%)	0.013
Admitted to an ICU	21 (34.4%)	18 (62.1%)	3 (9.4%)	<0.001
Surgical operations	9 (14.8%)	6 (20.7%)	3 (9.4%)	0.287
Chronic dialysis	3 (4.9%)	2 (6.9%)	1 (3.1%)	0.600
Received any antibiotics within 3 months	34 (55.7%)	21 (69.0%)	14 (43.8%)	0.048
Cephalosporins	21 (34.4%)	12 (41.4%)	9 (28.1%)	0.277
Penicillins	1 (1.6%)	1 (3.4%)	0 (0.0%)	0.475
Carbapenems	12 (19.7%)	8 (27.8%)	4 (12.5%)	0.139
Fluoroquinolones	7 (11.5%)	6 (20.7%)	1 (3.1%)	0.046
Beta-lactams/beta-lactamase inhibitors	13 (21.3%)	9 (31.0%)	4 (12.5%)	0.078
Glycopeptides	2 (3.3%)	1 (3.4%)	1 (3.1%)	1.000

### Multilocus sequence typing of CRKp strains

**Fig 1:**
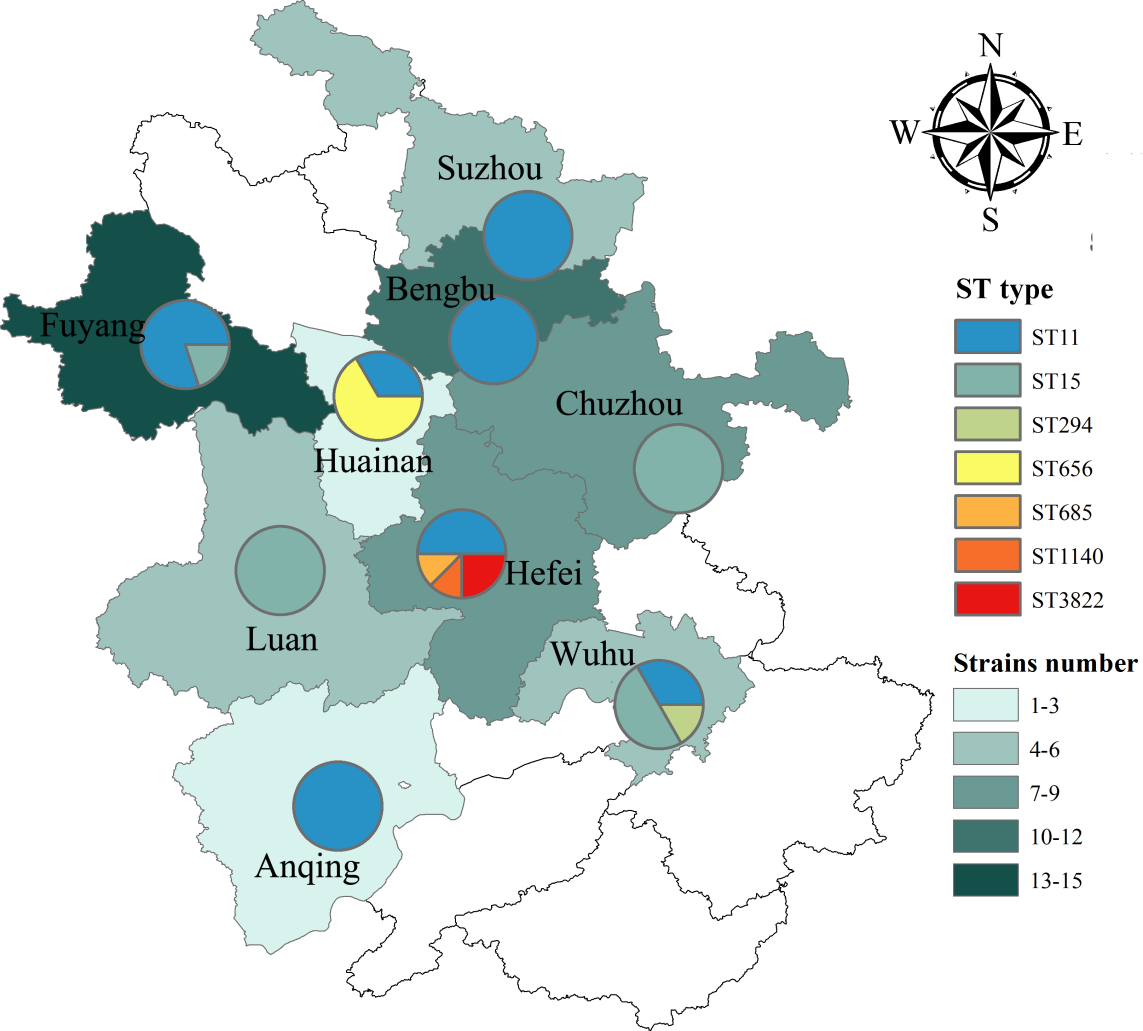
Distribution of STs of 61 *CRKp* strains in different regions in Anhui Province. The map is derived from the public standard map website (http://bzdt.ch.mnr.gov.cn/).

**Fig 2:**
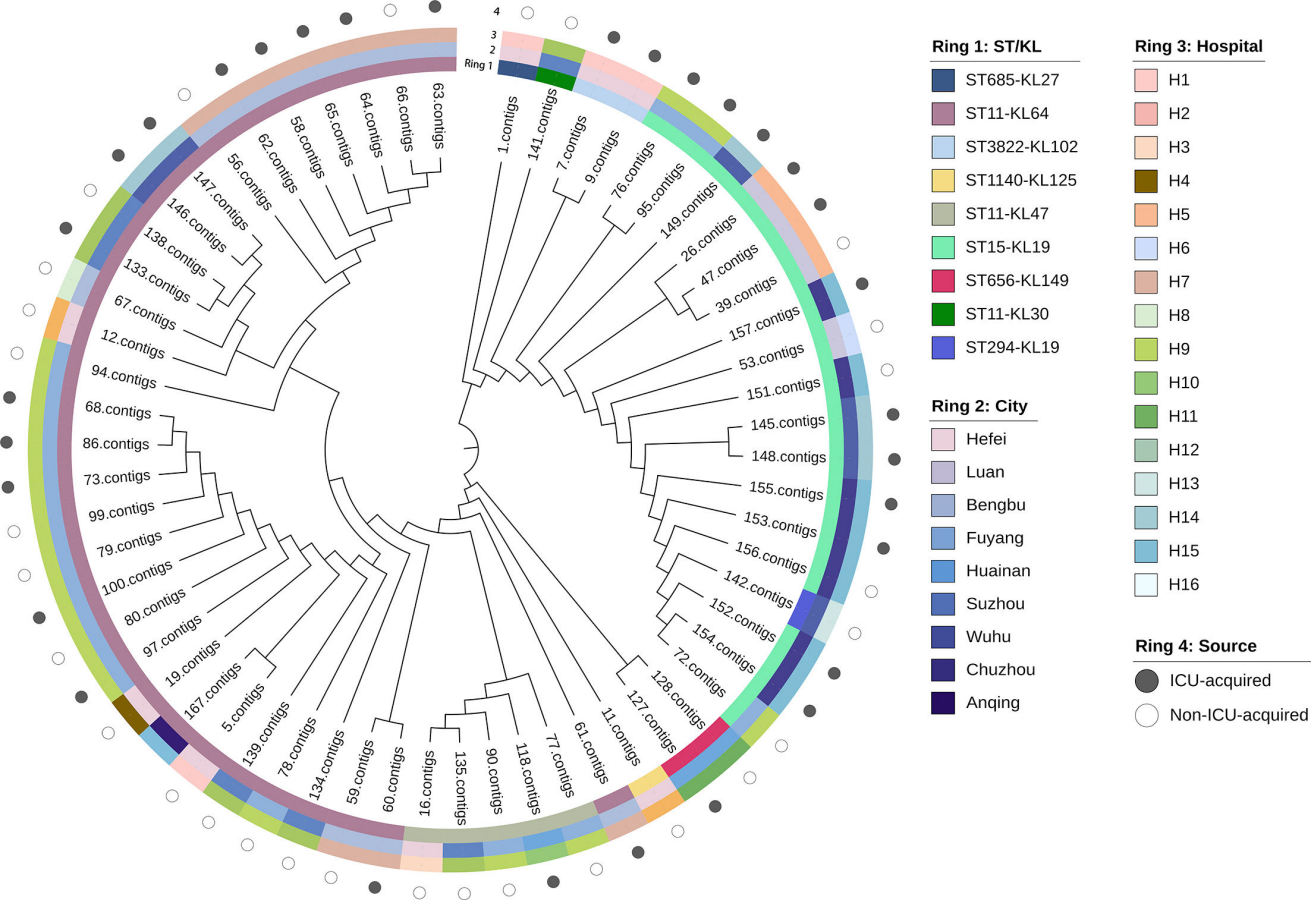
Characteristics of 61 *CRKp* strains in this study. The core genome phylogenetic tree was constructed by the neighbor-joining method.

In total, seven different STs were identified in 61 *CRKp* strains. In terms of geographical area, ST11 was detected in most participating cities, but was mainly concentrated in the northern cities (i.e., Bengbu, Fuyang, and Suzhou) of Anhui Province, while ST15 was mainly concentrated in the central and southern cities (i.e., Chuzhou, Luan, and Wuhu) (Fig. 1). As shown in Table 2, ST11 was the most dominant, accounting for 60.7% of all strains, followed by ST15 (27.9%). ST11 was consistently common among strains from the non-ICU-acquired group (69.0%) and ICU-acquired group (53.1%). In the ST11 strains, KL64 (83.8%) was the predominant capsular type, followed by KL47 (13.5%), while KL30 (2.7%) was relatively rare (Table S2). Furthermore, KL19 was the unique capsular type in ST15 strains. ST11-KL64 was the most common among the 61 strains, followed by ST15-KL19, with both strains being associated with different cities and hospitals (Fig. 2). ST15 and ST656 were both detected in strains from the non-ICU-acquired group and the ICU-acquired group. In comparison, ST294, ST685, and ST1140 were detected only in the non-ICU-acquired group, while ST3822 was only detected in the ICU-acquired group.

**TABLE 2:** Sequence types (STs) of 61 *CRKp*

Sequence type	Total (*N* = 61)	Non-ICU-acquired *CRKp* (*n* = 29)			ICU-acquired *CRKp* (*n* = 32)		
		Infection	Colonization	Total	Infection	Colonization	Total
ST11	37 (60.7%)	8 (61.5%)	12 (75.0%)	20 (69.0%)	6 (50.0%)	11 (55.0%)	17 (53.1%)
ST15	17 (27.9%)	3 (23.1%)	2 (12.5%)	5 (17.2%)	5 (41.7%)	7 (35.0%)	12 (37.5%)
ST656	2 (3.3%)	0 (0.0%)	1 (6.3%)	1 (3.4%)	0 (0.0%)	1 (5.0%)	1 (3.1%)
ST3822	2 (3.3%)	0 (0.0%)	0 (0.0%)	0 (0.0%)	1 (8.3%)	1 (5.0)	2 (6.3%)
ST294	1 (1.6%)	1 (7.7%)	0 (0.0%)	1 (3.4%)	0 (0.0%)	0 (0.0%)	0 (0.0%)
ST685	1 (1.6%)	1 (7.7%)	0 (0.0%)	1 (3.4%)	0 (0.0%)	0 (0.0%)	0 (0.0%)
ST1140	1 (1.6%)	0 (0.0%)	1 (6.3%)	1 (3.4%)	0 (0.0%)	0 (0.0%)	0 (0.0%)

### Carbapenemase and virulence-associated genes

**Fig 3:**
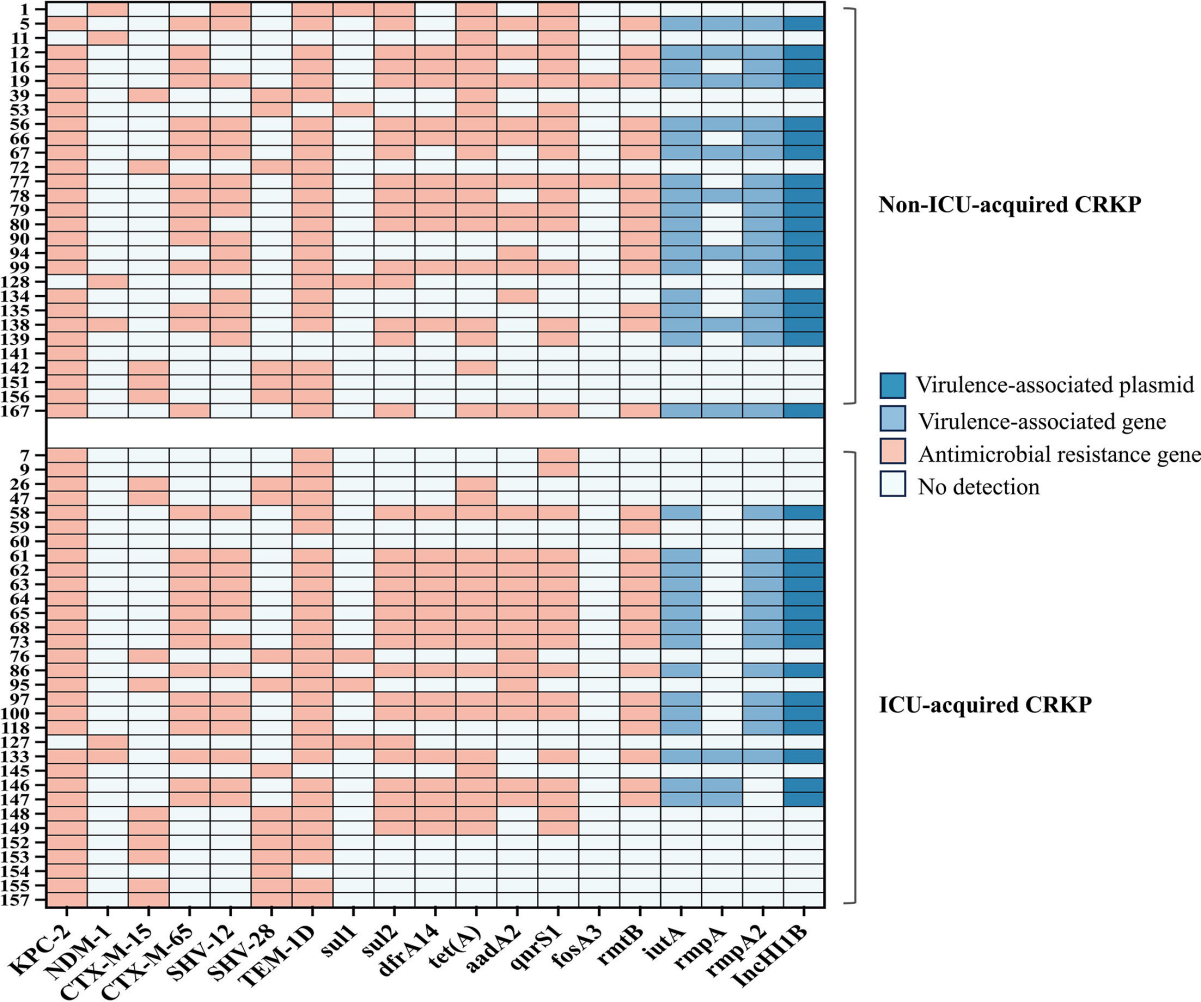
Resistance and virulence-associated genes of 61 *CRKp* strains.

All of the analyzed strains carried 1–11 resistance genes across several commonly used drug classes in China including *β*-lactams (*bla*KPC-2, *bla*NDM-1, *bla*CTX-M-15, *bla*CTX-M-65, *bla*SHV-12, *bla*SHV-28, *bla*TEM-1D), sulfonamide (*sul1*, *sul2*), trimethoprim (*dfrA14*), tetracycline (*tet(A*)), aminoglycosides (*aadA2*, *rmtB*), quinolones (*qnrS1*), and fosfomycin (*fosA3*); Fig. 3). According to the carbapenemase gene analysis data, 93.4% (57/61) of the *CRKp* strains carried the *bla*KPC-2 gene, 9.8% (6/61) carried the *bla*NDM-1 gene, and two strains both carried the *bla*KPC-2 and the *bla*NDM-1 genes. No other carbapenemase genes were detected. A high abundance of virulence genes (55.7%, 34/61) including *iutA* (55.7%, 34/61), *rmpA* (18.0%, 11/61), and *rmpA2* (52.5%, 32/69) were identified, with *iutA +rmpA2* (37.7%, 23/61) being the most common combination, followed by *iutA +rmpA + rmpA2* (14.8%, 9/61) and *iutA +rmpA* (3.3%, 2/61). In addition, the IncHI1B plasmid was found in all of the 34 strains carrying the detected virulence genes.

### CgMLST analysis of 61 CRKp strains

**Fig 4:**
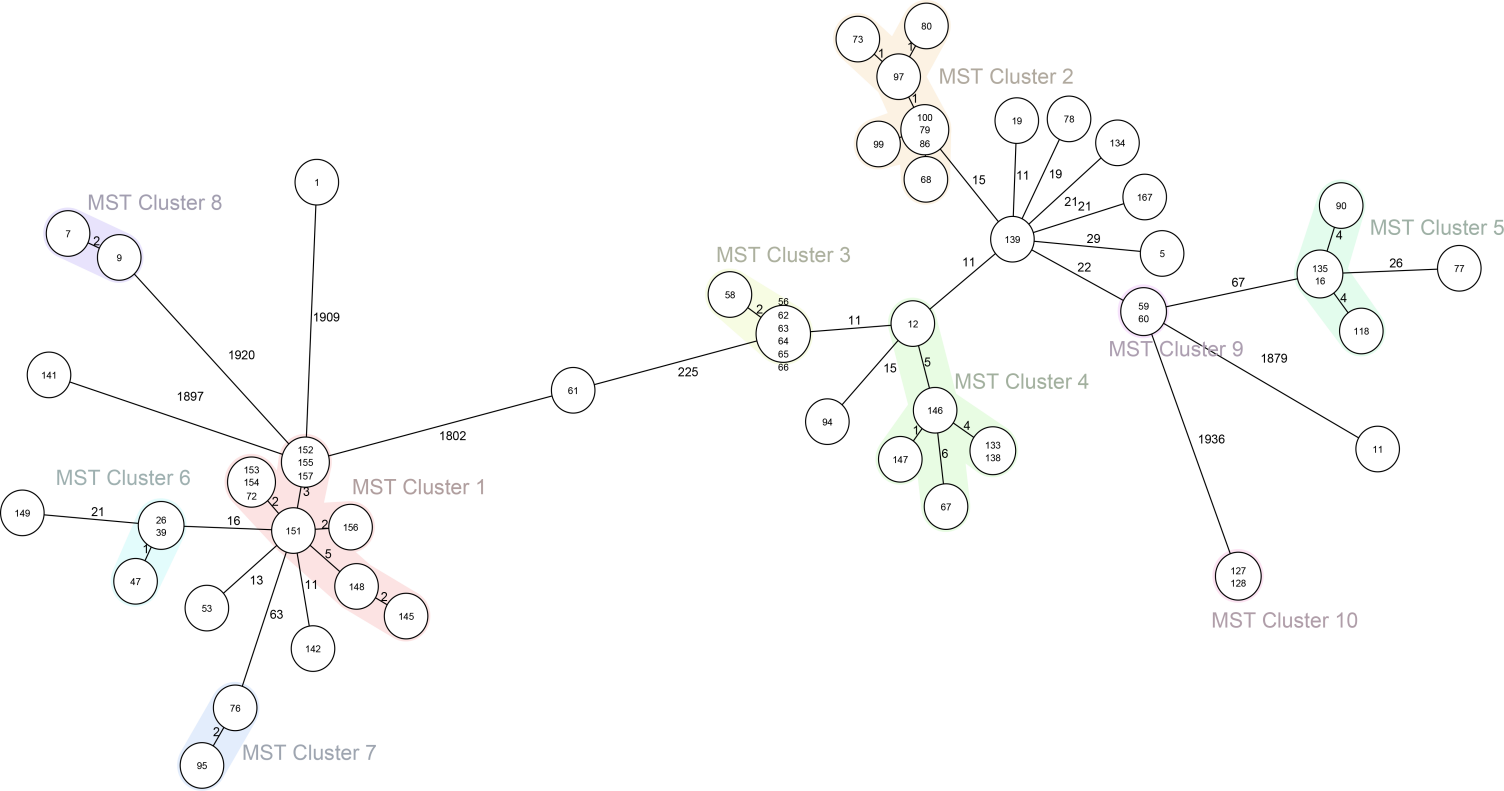
Ridom SeqSphere + minimum spanning tree (MST) for 61 *CRKp* strains based on 2,358 target genes with pairwise ignoring missing values. MST Cluster distance threshold: 10.

Phylogenetic analysis based on cgMLST analysis with a cutoff of 10 allele differences resulted in 10 clusters (Fig. 4). Multiple highly homogeneous *CRKp* strains were identified between cities in Anhui Province. For example, 10 strains (No.72, No.145, No.148, and No.151–157) in Cluster 1 were collected from three hospitals in different cities and presented 0 to 5 allelic differences, while four strains in Cluster 5 were collected from four hospitals in different cities and presented 0 to 4 allelic differences. Moreover, several larger related clusters were also detected in the same hospitals by cgMLST analysis, and potential intrahospital transmission of *CRKp* was hypothesized. For example, eight strains in Cluster 2 (No.68, No.73, No.79, No.80, No.86, No.97, No.99, and No.100) harboring ST11-KL64 collected from different patients in the same hospital (H9) in Fuyang city were clonally clustered and presented 0 to 1 allelic differences. Seven strains (No.56, No.58, and No.62–66) in Cluster 3 harboring ST11-KL64 collected from different patients in the same hospital (H7) in Bengbu city were clonally clustered and presented 0 to 2 allelic differences. In addition, two ICU-acquired *CRKp* strains (No.59 and No.60) harboring ST11-KL64 in Cluster 9 collected from one hospital (H7) in Bengbu city showed no allelic difference. Two *CRKp* strains (No.127 and No.128) harboring ST656-KL149 in Cluster 10 collected from one hospital (H11) in Huainan City also showed no allelic difference. Importantly, *CRKp* strains No.127 and No.128 were identified as cases of ICU-acquired and non-ICU-acquired colonization, respectively, which supports the hypothesis that colonization is a major risk factor for *CRKp* transmission.

## DISCUSSION

In this multicenter study, we applied WGS to describe the genomic characteristics and phylogenetic relatedness of colonization and infection with *CRKp* in ICU patients. The data generated in this work will further aid the understanding of the prevalence and genetic diversity of *CRKp* in epidemic regions. Carbapenemase-mediated resistance, specifically to *CRKp*, poses a serious threat to global public health. *K. pneumoniae* was first reported to produce *bla*KPC-2 carbapenemase in 2007 in China, and since then *CRKp* has been extensively reported with the number of incidences continuing to increase at a rapid pace throughout the country (34). ICUs are considerable hubs for the emergence and transmission of antimicrobial resistance (35). Herein, findings show that non-ICU-acquired *CRKp* strains are significantly (*P* < 0.05) more likely to be isolated from patients who have been transferred from another hospital, admitted to a hospital or an ICU within 1 year, and have received any antibiotics or fluoroquinolones within 3 months, compared to individuals with ICU-acquired *CRKp*. These findings can help identify patients who are at high risk of carrying *CRKp* at ICU admission for timely clinical identification and for implementing effective infection prevention and control strategies.

The use of WGS in the surveillance and control of antimicrobial resistance has been a major development that now allows for early detection and tracking of transmission, as well as thorough characterization of pathogens and resistance mechanisms (36). MLST data revealed that ST11 (97.3% carrying KL64/KL47) was the dominant type from the non-ICU-acquired group and the ICU-acquired group, which further increased the body of evidence suggesting that sister clades of ST11 carrying either the KL64 or KL47 capsular type were the predominant *CRKp* clones in China and that there are differences in prevalence between China and other countries (37). A recent study from Northeast China indicated that the dominant ST type of *CRKp* in bloodstream infections changed from ST11 to ST15 (38). Notably, findings from the present study revealed more than a onefold increase in the frequency of ST15 among the ICU-acquired group (37.5%) compared to the non-ICU-acquired group (17.2%). This highlights the need for additional development of effective control measures and continued monitoring. Moreover, other types, including ST656, ST3822, ST294, ST685, and ST1140, were detected in different cities in Anhui Province. These types differ from those identified in a multicenter cross-sectional study in Henan Province, China (39), implying the genetic diversity of *CRKp* in different regions of China.

In this study, 93.4% of the *CRKp* strains were shown to harbor the *bla*KPC-2 gene, which was consistent with findings of previous studies showing a high prevalence of *bla*KPC-2 in *CRKp* in ICUs and hospitalized patients in China (4, 39), which was higher than the prevalence rates reported in South America, the USA, Australia, Lebanon, and Singapore (4). The prevalence of the *bla*NDM-1 gene was 9.8%, and no other *bla*NDM genes were detected. This relatively low prevalence of the *bla*NDM gene in *CRKp* was similar to that reported in China, South America, and the USA in a multicenter cohort study (4); however, this prevalence was lower than the reported 28.6% for *bla*NDM in a single-center study in Ganzhou, China and 23.0% in Australia, Lebanon, and Singapore (4, 40). Moreover, results showed that the high prevalence (55.7%, 34/61) of virulence genes including *iutA*, *rmpA*, and *rmpA2* among *CRKp* strains, which was greater than the 34.2% positive rate reported in a multicenter study from China (41). Moreover, the IncHI1B plasmid was found in all of the 34 strains carrying the detected virulence genes, which was consistent with a previous study that found that the IncHI1B plasmid was predominantly responsible for the detected virulence genes in *CRKp* (42). Together with existing evidence suggesting that most *CRKp* strains harboring virulence genes are identified to be hypervirulent, the hypervirulent *CRKp* strains are now recognized as being an important entity in cases of *CRKp* colonization and infection in China (41). These findings highlighted regional differences in the prevalence of carbapenemase and virulence-associated genes of *CRKp*, which may be caused by local clonal expansions (37).

Phylogenetic analysis based on cgMLST analysis revealed 10 clusters among the 61 *CRKp* strains. Multiple highly homogeneous *CRKp* strains were identified from the same hospital as well as different hospitals, which suggests the potential interregional and intrahospital spread of *CRKp*, implying major challenges for the prevention and control of *CRKp* in healthcare facilities. Similarly, rapid interregional and interhospital transmission has been documented for NDM-producing *CRKp* (43). One previous study has indicated that high-risk clones in healthcare facilities have driven the spread of *CRKp* worldwide (44). In addition, our data also demonstrated that closely related *CRKp* strains were shared between different patients with non-ICU- and ICU-acquired colonization, which highlighted the importance of asymptomatic colonization in the spread of *CRKp* (17). These geographical diversities and the relevance of the genetic background of the *CRKp* strains likely reflect the widespread distribution mixed with locally specific strains in different regions of Anhui Province.

A limitation of this study is that despite this being a multicenter study design, other districts in China were not included and the study period might be relatively short for collecting isolates. Another limitation is that the links between genomic characteristics and patient outcomes were not analyzed in the present study. In addition, the exact route of clonal transmission and horizontal transfer of resistance and virulence genes in *CRKp* could not be confirmed without further data.

This prospective multicenter study revealed the widespread distribution of *CRKp* strains in combination with locally specific strains in different regions of Anhui Province. The potential of interregional and intrahospital spread of *CRKp* was hypothesized. Given the high prevalence of virulence genes, there is an urgent need for more effective infection monitoring and transmission control strategies. In addition, the role of asymptomatic colonization in *CRKp* transmission should be further investigated. Findings highlight the need for coordinated efforts between healthcare facilities and networks to allow for more effective and precise control strategies for *CRKp* spread, with WGS being a helpful tool that could aid in this goal.

## Data Availability

The genome sequences of the 61 strains have been deposited in the National Center of Biotechnology Information (NCBI) database under the BioProject accession number PRJNA1171938.

## SUPPLEMENTAL MATERIAL

Supplemental tables spectrum.01584-24-s0001.doc Table S1 and S2.

ASM does not own the copyrights to Supplemental Material that may be linked to, or accessed through, an article. The authors have granted ASM a non-exclusive, world-wide license to publish the Supplemental Material files. Please contact the corresponding author directly for reuse.
