# Supplementary material for: Genomic characteristics and phylogenetic analyses of colonization and infection with carbapenem-resistant Klebsiella pneumoniae in multicenter intensive care units: a cohort study
Source: Microbiol Spectr. 2025 Feb 24;13(4):e01584-24. doi: 10.1128/spectrum.01584-24 (PMC11960118; doi:10.1128/spectrum.01584-24)
Supplement: Supplemental tables — Table S1 and S2. [file spectrum.01584-24-s0001.doc]

**Table S1** Information on sources of 61 recovered *CRKp*

| No of isolates | Study facility | |  | Patient | |  | Non-ICU-acquired | |  | ICU-acquired | |
| --- | --- | --- | --- | --- | --- | --- | --- | --- | --- | --- | --- |
|  | No of hospital | Category |  | Sex | Age (y) |  | Infection | Colonization |  | Infection | Colonization |
| 1 | H1 | Provincial |  | Female | 52 |  | Yes |  |  |  |  |
| 5 | H1 | Provincial |  | Male | 29 |  |  | Yes |  |  |  |
| 7 | H1 | Provincial |  | Male | 49 |  |  |  |  | Yes |  |
| 9 | H1 | Provincial |  | Male | 56 |  |  |  |  |  | Yes |
| 11 | H2 | Provincial |  | Male | 46 |  |  | Yes |  |  |  |
| 12 | H2 | Provincial |  | Male | 47 |  |  | Yes |  |  |  |
| 16 | H3 | Provincial |  | Male | 67 |  |  | Yes |  |  |  |
| 19 | H4 | Municipal |  | Male | 55 |  |  | Yes |  |  |  |
| 26 | H5 | Municipal |  | Male | 65 |  |  |  |  |  | Yes |
| 39 | H5 | Municipal |  | Male | 70 |  | Yes |  |  |  |  |
| 47 | H5 | Municipal |  | Male | 48 |  |  |  |  |  | Yes |
| 53 | H6 | District |  | Male | 74 |  |  | Yes |  |  |  |
| 56 | H7 | Municipal |  | Male | 27 |  | Yes |  |  |  |  |
| 58 | H7 | Municipal |  | Male | 65 |  |  |  |  |  | Yes |
| 59 | H7 | Municipal |  | Male | 83 |  |  |  |  | Yes |  |
| 60 | H7 | Municipal |  | Male | 41 |  |  |  |  |  | Yes |
| 61 | H7 | Municipal |  | Male | 46 |  |  |  |  | Yes |  |
| 62 | H7 | Municipal |  | Female | 74 |  |  |  |  | Yes |  |
| 63 | H7 | Municipal |  | Female | 87 |  |  |  |  |  | Yes |
| 64 | H7 | Municipal |  | Female | 45 |  |  |  |  |  | Yes |
| 65 | H7 | Municipal |  | Male | 86 |  |  |  |  |  | Yes |
| 66 | H7 | Municipal |  | Male | 68 |  |  | Yes |  |  |  |
| 67 | H8 | Municipal |  | Male | 74 |  | Yes |  |  |  |  |
| 68 | H9 | Municipal |  | Male | 66 |  |  |  |  |  | Yes |
| 72 | H9 | Municipal |  | Male | 73 |  |  | Yes |  |  |  |
| 73 | H9 | Municipal |  | Male | 75 |  |  |  |  |  | Yes |
| 76 | H9 | Municipal |  | Female | 63 |  |  |  |  |  | Yes |
| 77 | H9 | Municipal |  | Male | 54 |  |  | Yes |  |  |  |
| 78 | H9 | Municipal |  | Female | 56 |  | Yes |  |  |  |  |
| 79 | H9 | Municipal |  | Female | 68 |  | Yes |  |  |  |  |
| 80 | H9 | Municipal |  | Female | 52 |  |  | Yes |  |  |  |
| 86 | H9 | Municipal |  | Female | 77 |  |  |  |  | Yes |  |
| 90 | H9 | Municipal |  | Male | 44 |  | Yes |  |  |  |  |
| 94 | H9 | Municipal |  | Male | 79 |  |  | Yes |  |  |  |
| 95 | H9 | Municipal |  | Female | 65 |  |  |  |  | Yes |  |
| 97 | H9 | Municipal |  | Male | 52 |  |  |  |  | Yes |  |
| 99 | H9 | Municipal |  | Female | 77 |  | Yes |  |  |  |  |
| 100 | H9 | Municipal |  | Female | 78 |  |  |  |  |  | Yes |
| 118 | H10 | Municipal |  | Female | 63 |  |  |  |  |  | Yes |
| 127 | H11 | District |  | Male | 38 |  |  |  |  |  | Yes |
| 128 | H11 | District |  | Female | 66 |  |  | Yes |  |  |  |
| 133 | H12 | Municipal |  | Female | 85 |  |  |  |  |  | Yes |
| 134 | H12 | Municipal |  | Male | 75 |  |  | Yes |  |  |  |
| 135 | H12 | Municipal |  | Male | 87 |  |  | Yes |  |  |  |
| 138 | H12 | Municipal |  | Female | 77 |  | Yes |  |  |  |  |
| 139 | H12 | Municipal |  | Female | 73 |  |  | Yes |  |  |  |
| 141 | H12 | Municipal |  | Male | 92 |  | Yes |  |  |  |  |
| 142 | H13 | Municipal |  | Female | 68 |  | Yes |  |  |  |  |
| 145 | H14 | Municipal |  | Male | 50 |  |  |  |  |  | Yes |
| 146 | H14 | Municipal |  | Female | 80 |  |  |  |  | Yes |  |
| 147 | H14 | Municipal |  | Female | 80 |  |  |  |  |  | Yes |
| 148 | H14 | Municipal |  | Female | 74 |  |  |  |  | Yes |  |
| 149 | H14 | Municipal |  | Male | 85 |  |  |  |  |  | Yes |
| 151 | H15 | Municipal |  | Male | 74 |  | Yes |  |  |  |  |
| 152 | H15 | Municipal |  | Male | 61 |  |  |  |  | Yes |  |
| 153 | H15 | Municipal |  | Female | 68 |  |  |  |  |  | Yes |
| 154 | H15 | Municipal |  | Male | 90 |  |  |  |  |  | Yes |
| 155 | H15 | Municipal |  | Male | 72 |  |  |  |  | Yes |  |
| 156 | H15 | Municipal |  | Male | 54 |  | Yes |  |  |  |  |
| 157 | H15 | Municipal |  | Female | 55 |  |  |  |  | Yes |  |
| 167 | H16 | Municipal |  | Male | 50 |  |  | Yes |  |  |  |

**Table S2** Distribution of capsule serotyping according to sequence types (STs) of 61 *CRKp*

| Capsule serotyping | Sequence type | | | | | | |
| --- | --- | --- | --- | --- | --- | --- | --- |
|  | ST11 | ST15 | ST656 | ST3822 | ST294 | ST685 | ST1140 |
| KL64 | 31 (83.8%) | 0 (0.0%) | 0 (0.0%) | 0 (0.0%) | 0 (0.0%) | 0 (0.0%) | 0 (0.0%) |
| KL47 | 5 (13.5%) | 0 (0.0%) | 0 (0.0%) | 0 (0.0%) | 0 (0.0%) | 0 (0.0%) | 0 (0.0%) |
| KL30 | 1 (2.7%) | 0 (0.0%) | 0 (0.0%) | 0 (0.0%) | 0 (0.0%) | 0 (0.0%) | 0 (0.0%) |
| KL19 | 0 (0.0%) | 17 (100%) | 0 (0.0%) | 0 (0.0%) | 1 (100.0%) | 0 (0.0%) | 0 (0.0%) |
| KL149 | 0 (0.0%) | 0 (0.0%) | 2 (100.0%) | 0 (0.0%) | 0 (0.0%) | 0 (0.0%) | 0 (0.0%) |
| KL102 | 0 (0.0%) | 0 (0.0%) | 0 (0.0%) | 2 (100.0%) | 0 (0.0%) | 0 (0.0%) | 0 (0.0%) |
| KL27 | 0 (0.0%) | 0 (0.0%) | 0 (0.0%) | 0 (0.0%) | 0 (0.0%) | 1 (100.0%) | 0 (0.0%) |
| KL125 | 0 (0.0%) | 0 (0.0%) | 0 (0.0%) | 0 (0.0%) | 0 (0.0%) | 0 (0.0%) | 1 (100.0%) |
| Total | 37 (100.0) | 17 (100%) | 2 (100.0%) | 2 (100.0%) | 1 (100.0%) | 1 (100.0%) | 1 (100.0%) |
